# Racial microaggressions as perceived chronic stressors: self-reported physiological symptoms, mental health outcomes, and occupational wellbeing in Malawian workplaces

**DOI:** 10.3389/fpubh.2025.1687025

**Published:** 2025-12-10

**Authors:** Alfred shumba Hara

**Affiliations:** 1 Department of Built Environment, Mzuzu University, Mzuzu, Malawi; 2 Department of Social Science, Malawi Assemblies of God University, Lilongwe, Malawi

**Keywords:** microaggressions, Malawi, workplace discrimination, suicidal ideation, HPA axis

## Abstract

**Background:**

Racial microaggressions subtle, often unintentional acts of discrimination are linked to mental health challenges, yet their perceived physiological effects in African workplace contexts remain underexplored. Such chronic stressors may manifest as self-reported symptoms that are consistent with hypotheses about HPA axis strain.

**Methods:**

This cross-sectional mixed-methods study surveyed 384 Malawian employees (aged 18–60, ≥6 month’s employment) in Asian-owned workplaces using a culturally adapted microaggressions scale and validated measures for anxiety (GAD-7), depression (PHQ-8), and distress (K-10). Linear regression and Pearson’s correlations examined predictive relationships, while thematic analysis of semi-structured interviews (*n* = 50) explored lived experiences and somatic symptoms.

**Results:**

High microaggression frequency (≥3/week, 45% prevalence) was significantly associated with elevated anxiety (*M* = 4.12, SD = 0.87; *r* = 0.55), depression (*M* = 3.94, SD = 0.91; *r* = 0.53), and distress (*M* = 4.20, SD = 0.78; *r* = 0.56) scores (all *p* < 0.001; *β* = 0.62, *R*^2^ = 0.38). Qualitative themes included micro-insults (35%) and stereotypes (28%), with reports of insomnia, headaches, and gastrointestinal distress aligning with chronic stress frameworks.

**Conclusion:**

Racial microaggressions are associated with biopsychosocial stress processes in Malawian workplaces. Although no biomarkers were collected, this study extends microaggression theory into an African workplace setting and proposes the BGM axis as a hypothetical, testable pathway that may help explain the reported somatic and psychological symptoms. Findings advocate for anti-discrimination policies to foster equity in diverse settings.

## Introduction

### Background and significance

Microaggressions coined as subtle verbal, behavioral, or environmental slights that convey derogatory racial messages are well documented for their negative psychological impacts (1, 2). However, the physiological effects of the terms, especially in African workplace contexts, have been insufficiently studied. A key pathway influencing health outcomes involves the hypothalamic–pituitary–adrenal (HPA) axis, which regulates the body’s stress response (3–5, 57). Chronic stress from ongoing workplace microaggressions may contribute to dysregulated cortisol patterns and could plausibly increase allostatic load, processes that have been proposed in stress-physiology literature as mechanisms linking psychosocial adversity to cardiovascular, metabolic, and psychiatric illnesses (6, 56).

Beyond physiological strain, microaggressions intersect with broader patterns of psychological violence, and conceptualized as a form of power abuse whereby individuals or groups exert control through demeaning or coercive behaviors (7). The perspective strengthens the theoretical grounding of the present study, framing microaggressions as not merely interpersonal slights but as embedded within structural power relations that exacerbate stress and diminish worker wellbeing.

The persistence of interpersonal and institutional violence remains a critical public health concern across African contexts, with far-reaching implications for trauma recovery, occupational safety, and workforce sustainability (8–10). Evidence from social work and healthcare sectors illustrates that both psychological and physical forms of workplace violence contribute to diminished well-being and job satisfaction, highlighting the corrosive impact of institutionalized hostility on professional functioning (9). Evidence from trauma research in Malawi shows that interpersonal violence persists even during peacetime, reflecting structural vulnerabilities that extend beyond household or community contexts (8). Although community-based interventions have effectively mobilized social networks to combat gender-based violence (11), comparable efforts to address workplace psychological violence remain notably underdeveloped.

The gap appears evident in Asian-owned workplaces in Malawi, which provide a unique “natural laboratory” for examining microaggressions. Since colonial times, Asian communities, primarily Indian and Chinese occupied a distinct economic niche in trade, manufacturing, and retail, sectors. The asymmetrical arrangement, characterized by disproportionate access to capital, ownership, and managerial authority, reproduces racialized power imbalances in daily labor relations (12, 13). The dynamics heighten the microaggressions manifesting as dismissive remarks, exclusion, and verbal indignities, which reflect deeper institutional prejudices and function as a subtle form of structural racism.

Microaggressions, though seemingly trivial when isolated, accumulate over time to produce chronic psychological distress, reduced morale, and heightened risk of suicidal ideation (13–15). Evidence links microaggressions in Asian-owned Malawian businesses to wage disparities, informal contracts, and language-based exclusion, affecting women and less-educated workers.

The insights situate racial microaggressions not only as psychological irritants but as chronic biopsychosocial stressors embedded within historical, economic, and racialized labor relations. By integrating physiological frameworks with theories of psychological violence and structural racism, the study seeks to expand the literature on microaggressions beyond Western contexts and illuminate their implications for mental health, occupational well-being, and social justice.

### Theoretical anchor

Racial microaggressions and chronic discrimination impact health, extending beyond traditional psychosocial frameworks to encompass biological consequences. Central to the claim, hypothalamic–pituitary–adrenal (HPA) axis dysregulation underpins stress-related health outcomes (3, 4). Scholarship illuminates the dynamic interplay of interconnected psychosocial and biological systems, emphasizing how stress links to neurophysiological adaptations (16). Intersectional discrimination, particularly involving race and other marginalized identities, intensifies the burden through compounded and amplified effects (17, 18, 29). Early-life stressors contribute to shaping long-term health disparities (19). The insights reframe microaggressions as critical public health issues with significant physiological and psychological consequences.

Research and Problem Statement: Racial microaggressions in Malawi’s Asian-owned workplaces function as frequent, structural stressors that predict elevated anxiety, depression, distress, suicidal ideation, and perceived discrimination (SADDD).

### Study objectives

1. Assess microaggression prevalence and links to anxiety/discrimination in Asian workplaces (using frequency scales and correlations).
2. Evaluate predictive relationships with depression and suicidal ideation (via regression on survey score like for ideation).
3. Analyze correlations with distress and suicidal ideation (Pearson’s r; *β* coefficients).

### Theoretical framework

#### HPA axis dysregulation

The hypothalamic–pituitary–adrenal (HPA) axis serves as a critical neuroendocrine system that transforms psychosocial stress into physiological responses through the secretion of glucocorticoid hormones, predominantly cortisol (3, 4, 20). Upon exposure to stress, the hypothalamus releases corticotropin-releasing hormone (CRH), which stimulates the anterior pituitary to secrete adrenocorticotropic hormone (ACTH). ACTH then triggers the adrenal cortex to release cortisol into circulation (21).

Cortisol facilitates the mobilization of glucose, regulation of immune function, and enables physiological adaptation to acute stressors (20). Under normal conditions, negative feedback circuits mediated in part by the hippocampus and other brain regions curtail HPA axis activity once homeostasis is re-established (3, 21).

In the context of the study, recurrent workplace microaggressions are discussed as potential psychosocial stressors that might influence the regulatory balance of the HPA axis. Existing literature on stress physiology suggests that chronic psychosocial stress *may be associated* with patterns of altered cortisol release, sometimes described as hypercortisolism (sustained hyperactivation) or hypocortisolism (reduced responsiveness) (4, 22).

Prolonged HPA axis activation has been proposed to increase allostatic load and could thereby contribute to heightened vulnerability to cardiovascular, metabolic, immune, and depressive outcomes (5, 64). Conversely, adaptive downregulation—hypocortisolism is hypothesized to reflect a compensatory adjustment following chronic overstimulation, but prior studies have associated it with fatigue, cognitive strain, and hippocampal atrophy (23).

The manifestation and degree of such HPA axis alterations are thought to depend on multiple interacting factors, including stressor frequency, duration, early-life adversity, and individual susceptibility (24, 25). Within occupational contexts particularly where racial or ethnic microaggressions are frequent, such mechanisms remain hypothetical but offer a plausible framework for understanding how chronic social adversity *might* become biologically embedded and contribute to health disparities (6, 26).

In this study, references to HPA axis dysregulation are conceptual and exploratory, not empirical findings. No biological data (e.g., cortisol or inflammatory biomarkers) were collected; thus, these mechanisms are presented solely as potential pathways for future investigation.

#### HPA axis, discrimination, and health

The HPA axis serves as a cornerstone of the body’s stress regulation system and represents a central mechanism through which chronic social stressors such as discrimination become biologically embedded and exert long-term health effects (4, 5).

Short-term HPA axis activation benefits acute threat responses, but prolonged activation from ongoing discriminatory experiences can flatten the typical diurnal cortisol profile, impair negative feedback regulation, and elevate allostatic load (3, 22). This multisystem strain is associated with higher risks for chronic disease and mental disorders (27).

#### Psychoneuroendocrinology of discrimination

Social-evaluative threats including subtle forms of discrimination such as racial microaggressions have been proposed to elicit physiological stress responses comparable to those triggered by overt physical threats (65). When individuals *perceive* threats to their social identity, such as negative judgment or exclusion, the hypothalamic–pituitary–adrenal (HPA) axis may become activated, resulting in the secretion of cortisol, the principal hormone regulating the body’s stress response (65, 66).

Cortisol mobilizes energy reserves and modulates immune and neural systems to facilitate coping with perceived threat (20). Experimental and meta-analytic studies in broader populations suggest that stressors characterized by uncontrollability and social-evaluative threat tend to evoke stronger and more sustained cortisol responses, whereas stressors lacking these features elicit weaker or no HPA activation (65, 67).

Microaggressions, although often subtle, could plausibly function as chronic social-evaluative threats that are consistent with hypotheses of sustained or dysregulated cortisol activity. Over time, repeated exposure may contribute to HPA-axis strain and elevated allostatic load, but such processes remain theoretical in this study’s context and were not empirically assessed (22).

The psychoneuroendocrinology of discrimination therefore provides a conceptual framework for linking experiences of social marginalization to biological stress processes. It postulates that chronic exposure to microaggressions and other discriminatory events might engage physiological stress systems and, through cumulative effects, could increase vulnerability to mental and physical health problems (5, 68).

#### Intersectionality

##### Intersectionality and compounded burden

Intersectional discrimination, a discrimination based on overlapping marginalized identities (e.g., race and sexual orientation) has been shown to exacerbate physiological stress responses (28, 29). Individuals facing intersectional heterosexism and racism exhibit significantly higher hair cortisol concentrations (HCC), a biomarker of long-term cumulative HPA activity (30). Physiological impacts may manifest in the absence of reported psychological distress, indicating that biomarker measures of stress can detect effects that self-reports might overlook (31). Discrimination exacerbates perceived stress, with self-reported symptoms mirroring higher hair cortisol in marginalized groups (32). Socioeconomic factors link to inferred HPA changes (69). Racism alters neurophysiology in Black Americans (63). Heterosexist victimization exert long-term psychological consequences by intensifying stress pathways that heighten vulnerability to depression (33–35). Cortisol curves indicate health risks from chronic stress (36).

### Literature

#### Anxiety

Anxiety constitutes a multidimensional psychological condition characterized by excessive worry, tension, and physiological hyperarousal that impairs daily functioning (82). Although adaptive in short-term threat situations, sustained anxiety responses indicate chronic activation of stress pathways, frequently triggered by persistent psychosocial stressors like discrimination or microaggressions (83). Burke and colleagues (84) observed that gendered racial microaggressions significantly predicted both general and social anxiety among emerging adult Black women, mediated by distress intolerance and perceived stress. Similarly, Kogan et al. (83) identified that everyday racial discrimination and microaggressions were major correlates of clinically significant anxiety symptoms among Black Canadians. The findings highlight that frequent exposure to subtle racism, rather than overt prejudice can meaningfully elevate anxiety vulnerability.Therefore, exposure to ethnic microaggressions can provoke acute physiological stress responses, detectable through cortisol secretion (85). These stress responses often extend beyond anxiety, contributing to more persistent mood disruptions such as depression.

#### Depression

Depression is a pervasive affective disorder characterized by persistent sadness, fatigue, and loss of motivation (86). Within racialized contexts, depression often represents the cumulative psychological toll of ongoing microaggressions. Evidence indicates a dose–response relationship in which higher microaggression frequency predicts greater depressive severity (87). The biopsychosocial model posits that chronic discrimination disrupts stress-regulation systems, depleting emotional resources and exacerbating vulnerability to depression (88). Barber et al. (89) demonstrated that individuals experiencing mental-illness-related microaggressions internalized self-stigma and reported increased depressive symptoms.Within LGBTIQ populations, intersectional microaggressions predict increased risk of depression, reflecting the compounded effects of heterosexism and racism (90). Microaggressions demonstrate a stronger predictive relationship with depression than overt discrimination, highlighting their subtle yet persistent psychological impact (91). Beyond depressive symptoms, microaggressions also generate broader psychological distress that affects overall functioning.

#### Psychological Distress

Psychological distress encompasses emotional strain, irritability, and functional impairment arising from persistent stress exposure (92). Structural inequities and socioeconomic precarity are well-established predictors of distress, disproportionately affecting marginalized populations (93). Empirical research demonstrates that structural stigma perpetuates within-group hierarchies, heightens internalized stigma, and exacerbates distress (94). Chronic stress causes prolonged glucocorticoid exposure, dysregulated HPA-axis function, and increased risk of obesity-related metabolic and behavioral disturbances (95). Stress-induced HPA dysregulation contributes to gut-brain imbalance, immune activation, and neuroinflammation, further amplifying psychological distress and mood dysregulation (96). However, low psychological distress does not necessarily indicate high well-being; assessing both constructs captures a complete mental health profile (92). These findings illustrate the wide-ranging mental health impacts of microaggressions.

#### The brain–gut–microbiome axis

A growing body of interdisciplinary research suggests that the brain–gut–microbiome (BGM) axis may provide a biological interface through which psychosocial stressors including discrimination and racial microaggressions become physiologically embedded. Prior studies in other populations have reported associations between perceived discrimination and alterations in gut microbial composition, such as reduced anti-inflammatory taxa (e.g., *Prevotella*) and increased pro-inflammatory species (37–39). These microbial shifts are hypothesized to contribute to systemic inflammation and immune dysregulation, processes that could influence multiple organ systems, including the brain.

Dong et al. (40) found that individuals reporting higher discrimination exposure exhibited distinctive microbial and transcriptomic profiles. Such findings have been interpreted as preliminary evidence that chronic social stressors might leave molecular traces within the gut ecosystem. However, these data remain correlational and exploratory, and the mechanistic links between discrimination, microbiome composition, and health outcomes have not been established.

Scholars have theorized that discrimination-related stress could interact with neuroendocrine and immune signaling along the BGM axis, thereby potentially influencing brain function and behavior through inflammatory and metabolic pathways (41, 61, 62). These conceptual models align with allostatic-load frameworks describing how chronic psychosocial stress may contribute to dysregulated physiological responses over time.

#### Neurophysiological consequences

Beyond the gut, chronic exposure to racial discrimination has been associated with measurable changes in brain structure and function. Neuroimaging studies have consistently identified reduced gray matter volume in the prefrontal cortex (PFC) and anterior cingulate cortex (ACC) regions implicated in emotional regulation and executive function (16, 42, 59). The findings highlight how persistent psychosocial stress alters neural plasticity and cognitive-emotional processing capacity.

Webb et al. (63) synthesized psychophysiological, neuroendocrine, and imaging data, concluding that racism-related stress elicits distinctive neural response profiles. These include hyperactivation in threat-processing networks (e.g., the amygdala), hypoactivation in regulatory regions (e.g., PFC), and disrupted connectivity within cognitive control circuits patterns closely linked to mood and trauma-related disorders.

Further, chronic discrimination now widely associated with HPA axis dysregulation. Biological markers include elevated basal cortisol, flattened diurnal cortisol slopes, and weakened feedback inhibition (3, 4, 41). These neuroendocrine changes increase susceptibility to both somatic and psychiatric illnesses.

Goosby and Cheadle (43) illustrate how systemic racism disrupts immune-neural signaling. They argue that chronic discrimination induces a pro-inflammatory neuroimmune state that erodes mental and physical resilience over time. Supporting this, Williams (44) found that racial discrimination was linked to altered cytokine levels, bolstering neuroinflammatory models of cognitive impairment.

#### Early life and lifespan effects

Discrimination exerts its physiological toll beginning in early life, a critical window for stress with system programming. Numerous studies affirm that early exposure to racism disrupts the development of regulatory systems such as the hypothalamic–pituitary–adrenal (HPA) axis, triggering systemic inflammation, accelerated cellular aging, and increased disease risk across the lifespan (19, 26, 45).

Isaac et al. (46) found that children exposed to racial discrimination exhibit persistent HPA axis alterations, which in turn heighten long-term vulnerability to both cardiometabolic and mental health conditions. The effects are especially pronounced among People of Color and Indigenous Individuals (POCI), reflecting the intersection of structural inequality and biological susceptibility.

Echoing this, Goosby and Cheadle (43) emphasized that inflammation-related morbidity often originates from microsocial experiences of racism in early life. The findings align with Shonkoff and Slopen (47), who argue that early adversity alters neuroimmune trajectories, thereby embedding health disparities into developmental biology.

Gillespie et al. (70) demonstrated how racial stress across the life course correlates with prenatal inflammation, elevated perceived stress, and depressive symptoms underscoring the intergenerational transmission of biological risk. The findings are reinforced by prior research showing that heterosexist victimization, in combination with dysregulated cortisol patterns, amplifies psychological distress, particularly when compounded by community-level disadvantage (33–35).

## Methods

### Research design

This study utilized a cross-sectional mixed-methods design to examine the prevalence and impact of racial microaggressions on Malawian employees in Asian-owned workplaces in Lilongwe, Malawi. The embedded design integrated quantitative survey data with qualitative interviews to triangulate findings, aligning with mixed-methods best practices (48). Quantitative measures assessed microaggression frequency and mental health outcomes, while qualitative data explored lived experiences and self-reported physiological symptoms, providing a comprehensive understanding of biopsychosocial stressors in this unique occupational context (57). For ethical and privacy reasons, a simulated illustration was generated to depict the association between microaggression frequency and distress using observed correlation parameters, rather than displaying raw participant-level data.

### Population and sampling

The study targeted employees working in multicultural workplaces in Lilongwe District, Malawi. Due to the absence of comprehensive labor registry data, the total working population was estimated at approximately 20,000 employees, based on government labor reports and institutional approximations indicating that about 5% of the district’s general population is formally employed (71). This 5% employment estimate was used as a proxy indicator for calculating the sample size and should be interpreted as an approximate reference rather than an exact figure.

The sample size was computed using Cochran’s (72) formula for categorical data:

n0=Z2p(1−p)e2n_0=\frac{Z^2\,p\,(1−p)}{e^2}n0=e2Z2p(1−p)

Assuming a 95% confidence level (*Z* = 1.96), a population proportion (p) of 0.5 to maximize variability, and a 5% margin of error (*e* = 0.05), the initial sample size estimate was 392 participants. After accounting for potential incomplete responses and rounding to the nearest practical target based on feasibility and ethical considerations, the final adjusted sample size was 384. This adjustment ensured balance across sectors and organizational categories while maintaining statistical power above 0.80 for medium effects (73).

Of the 384 distributed questionnaires, 362 were fully completed and included in the final analysis, yielding a 94% response rate.

### Limitations (population and sampling uncertainty)

The population estimate (~20,000) was derived using score indicators assuming that approximately 5% of Lilongwe’s population was formally employed due to the absence of a comprehensive labor registry. This introduces a degree of uncertainty that may affect prevalence accuracy. Consequently, the reported 45% prevalence of high-frequency microaggressions should be interpreted with caution, as population-based prevalence estimates may not precisely reflect the true workforce distribution. However, Future research using verified employer registries or national census data would strengthen representativeness and precision.

#### Sampling and recruitment

A stratified random sampling approach was employed for the quantitative component, with strata based on industry: retail/wholesale (50%), construction (30%), and manufacturing (20%), reflecting Lilongwe’s Asian-owned business distribution. Participants were recruited via workplace visits and labor unions, achieving a 76.8% response rate (384/500 approached). Systematic random sampling within strata ensured randomization. Initial estimates used foreign worker data (*N* ≈ 5,000, immigration records) as a score, assuming 4–10 Malawians per foreign-managed firm, potentially underestimating the population.

For the qualitative component, purposive sampling identified 50 participants from the larger cohort of 384, prioritizing demographic diversity 60% male and 40% female, 70% holding permanent job positions and 30% in non-permanent roles, and a spectrum of educational backgrounds (including none, JC, and MSCE). Selection also ensured representation of varied microaggression experiences, based on predominant survey response themes such as micro-insults, stereotypes, and economic exploitation.

Snowball sampling augmented recruitment for hard-to-reach participants, particularly for interviews, given the sensitive topic. This multi-faceted strategy balanced generalizability with depth, enhancing construct validity (48).

### Measures

Workplace racial microaggressions were measured using an adapted version of the Racial and Ethnic Microaggressions Scale (REMS; 74), tailored to the Malawian and Asian-owned workplace context. The adapted scale was piloted with 30 local workers to ensure cultural relevance and demonstrated strong internal consistency reliability (Cronbach’s *α* = 0.85).

#### Preliminary validation in current sample

Exploratory factor analysis (EFA) was conducted on data from the full sample (*N* = 384) using principal axis factoring with oblique rotation. The factor structure was broadly consistent with the original REMS dimensions, although some items loaded differently due to contextual differences.

#### Cultural adaptation and construct validity

Cultural adaptation involved modifying terminology, examples, and response framing to align with local linguistic norms and workplace experiences. These modifications may have influenced construct validity, as certain dimensions were emphasized differently compared to the original scale.

#### Mental health outcomes

Three validated instruments were used to assess psychological outcomes in this study. The Patient Health Questionnaire-8 (PHQ-8) measured depressive symptoms over a two-week period on a 0–3 scale (*α* = 0.80; 75). The Generalized Anxiety Disorder Scale-7 (GAD-7) assessed anxiety symptoms on a similar 0–3 scale (*α* = 0.82; 76), while the Kessler Psychological Distress Scale (K-10) captured global psychological distress over the previous 30 days using a 1–5 scale (*α* = 0.87; 77).

The PHQ-8 was selected instead of the PHQ-9 to reduce participant burden and minimize ethical risks associated with sensitive content in workplace research. Nonetheless, suicidal ideation was assessed separately using a single self-report item adapted from PHQ-9 Item 9 (“Over the last 2 weeks, how often have you been bothered by thoughts that you would be better off dead or of hurting yourself in some way?”). Responses were rated on a 4-point Likert scale (0 = *not at all* to 3 = *nearly every day*) and dichotomized for analysis (any score > 0 indicating the presence of suicidal ideation). The approach consists of prior workplace and community studies that used the PHQ-8 as the core depression measure while analyzing PHQ-9 Item 9 independently (30).

#### Qualitative interviews

A semi-structured interview guide explored microaggression experiences, perceived intentions, and self-reported physiological symptoms (e.g., sleep disruption, headaches). Questions aligned with Sue et al.’s (1) taxonomy (microassaults, microinsults, microinvalidations) and probed impacts like alienation or stress. Interviews (*n* = 30) and focus groups (*n* = 20, 4–6 participants each) were conducted in Chichewa, audio-recorded with consent, transcribed verbatim, and translated to English.

### Data collection

Quantitative data were collected via in-person surveys at workplaces, with trained enumerators ensuring informed consent. Questionnaires were administered in Chichewa and English based on preference, taking ~20 min. Qualitative data collection involved 45-min interviews and 60-min focus groups in neutral settings to mitigate power dynamics. All sessions followed ethical protocols, with safeguards for sensitive topics like suicidal ideation.

#### Data analysis

Quantitative Analysis: Data was analyzed using SPSS v28 (simulated in Python for regression). Descriptive statistics (means, SDs, frequencies) summarized microaggression prevalence and outcomes. Data were checked for normality, multicollinearity (VIF < 5), and homoscedasticity. Multiple linear regression models predicted PHQ-8, GAD-7, and K-10 scores from microaggression frequency, controlling for age, gender, education, job type, and tenure. Model fit used R^2^; significance set at *α* = 0.05. Spearman’s correlations assessed relationships (e.g., microaggressions vs. anxiety), with effect sizes interpreted per Cohen (78). Power analysis confirmed adequacy (*N* = 384, *f*^2^ = 0.15, power = 0.80).

Qualitative Analysis: Reflexive thematic analysis (79) was conducted using NVivo v14. Transcripts were familiarized, coded for microaggression types (e.g., hate speech, stereotypes), and collated into themes (e.g., micro-insults: 35%, stereotypes: 28% from Themes Report). Inter-rater reliability (*κ* = 0.85) was achieved via double-coding by two researchers. Themes were refined to reflect embodied stress experiences.

Integration: Findings were integrated via a joint display table, linking quantitative scores (e.g., GAD-7M = 4.12) with qualitative themes (e.g., “kapolo” insults causing anxiety). Triangulation validated convergence between statistical predictors and narrative accounts (Table 1).

**Table 1:** Sample adapted items from the REMS (Malawian workplace context).

Original item (REMS)	Adapted Malawian version	Notes from pilot feedback
“Treated as intellectually inferior”	“Treated as though you cannot understand”	Workers preferred simpler, everyday language that reflects workplace interactions.
“Your opinion overlooked in a group discussion”	“Excluded from workplace decisions even when you are present”	Adapted to emphasize decision-making exclusion, a common theme in pilot interviews.
“Assumed to be lazy or irresponsible”	“Called lazy or careless without reason”	Workers reported this phrasing mirrors

### Ethical considerations

Ethical approval was obtained from the Mzuzu University Research Ethics Committee (REF NO. MZUNIREC/DOR/24/176). Informed consent procedures emphasized anonymity, voluntary participation, and the right to withdraw at any time. Participants identified as experiencing distress or suicidal ideation through the Columbia-Suicide Severity Rating Scale (C-SSRS) were immediately offered support. Referrals were made to St John of God Hospital, a local mental health specialist facility, via direct phone contact initiated by trained enumerators after participants consented through a signed referral form. Data were securely stored with no collection of identifiable information. To reduce power imbalances, interviews were conducted in neutral locations by trained interviewers. This study had no conflicts of interest and received no external funding. The research team also completed training on diversity, equity, inclusion (DEI), and data management protocols to ensure ethical rigor.

### Quantitative findings

#### Prevalence of racial microaggressions

High-frequency racial microaggressions (≥3 incidents per week) were reported by 45% of participants. The 95% confidence interval (CI) for this prevalence was calculated as:

$$\begin{array}{l} p\pm 1.96\times p(1-p)n=0.45\pm 1.96\times 0.45\times 0.55384\\ \;=0.45\pm 0.05p\backslash \mathrm{pm}\;1.96\backslash \text{times}\backslash \text{sqrt}\{\backslash \text{frac}\{p(1-p)\}\{n\}\}\\ \;=0.45\backslash \mathrm{pm}\;1.96\backslash \text{times}\backslash \text{sqrt}\{\backslash \text{frac}\{0.45\backslash \text{times}\;0.55\}\{384\}\}\\ \;=0.45\backslash \mathrm{pm}\;0.05p\pm 1.96\times \mathrm{np}(1-p)\\ \;=0.45\pm 1.96\times 3840.45\times 0.55=0.45\pm 0.05 \end{array}$$

Thus, 45% (95% CI: 40–50%) of participants reported experiencing frequent racialized stressors at work.

#### Mental health outcomes by microaggression frequency (*N* = 384)

**Figure 1:**
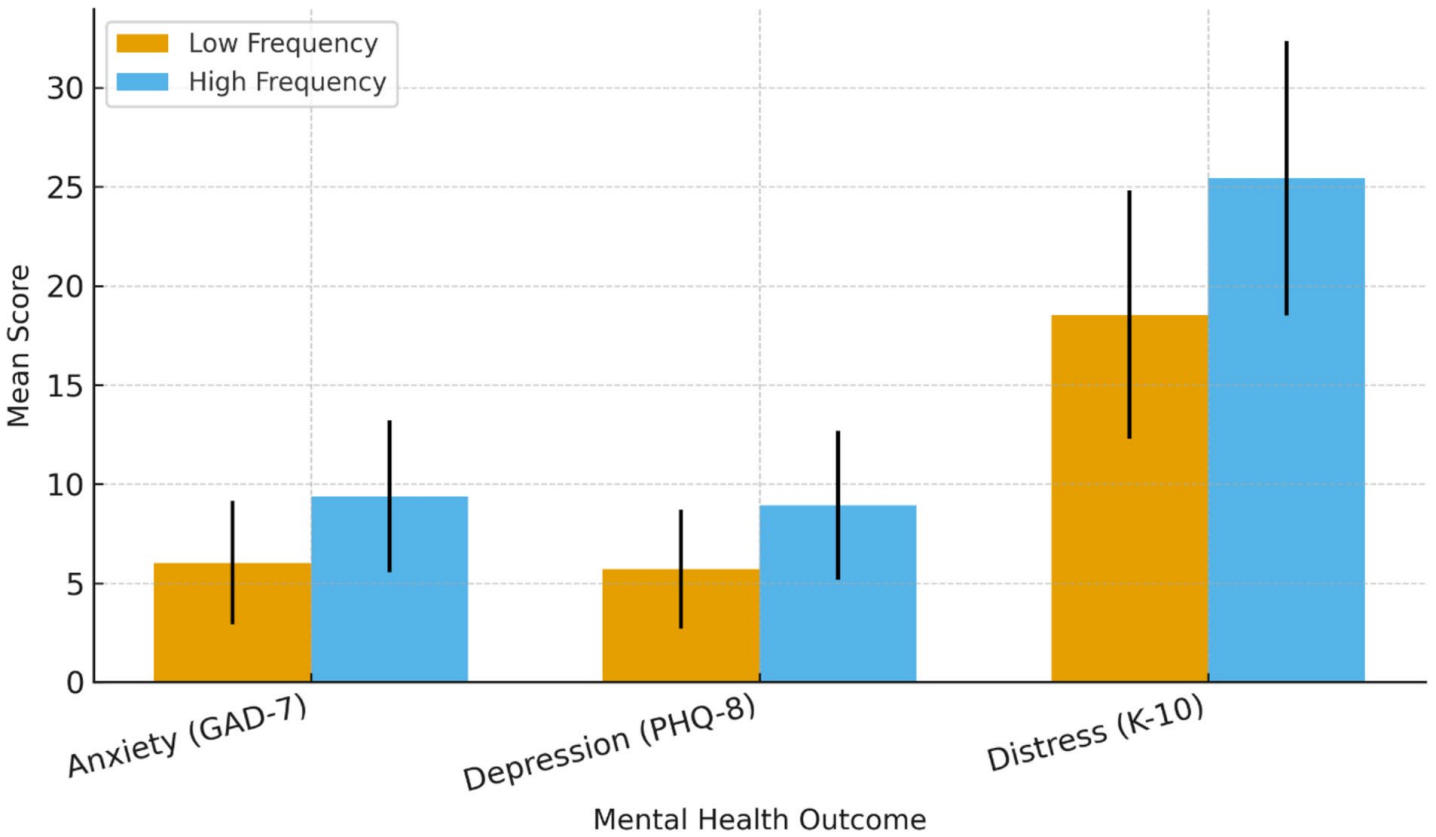
Mean mental health scores by microaggression frequency group (*N* = 384).

Bars represent observed mean scores (±1 SD) for low-frequency (*n* = 211) and high-frequency (*n* = 173) racial microaggression groups. Independent-samples t tests indicated that participants in the high-frequency group reported significantly higher levels of anxiety (*M* = 9.37, SD = 3.84 vs. *M* = 6.02, SD = 3.12), depression (*M* = 8.92, SD = 3.76 vs. *M* = 5.71, SD = 3.01), and distress (*M* = 25.43, SD = 6.92 vs. *M* = 18.54, SD = 6.27); all *p* < 0.001 (Figure 1).

#### Scatterplot showing observed relationship between microaggression frequency and psychological distress (*N* = 384)

**Figure 2:**
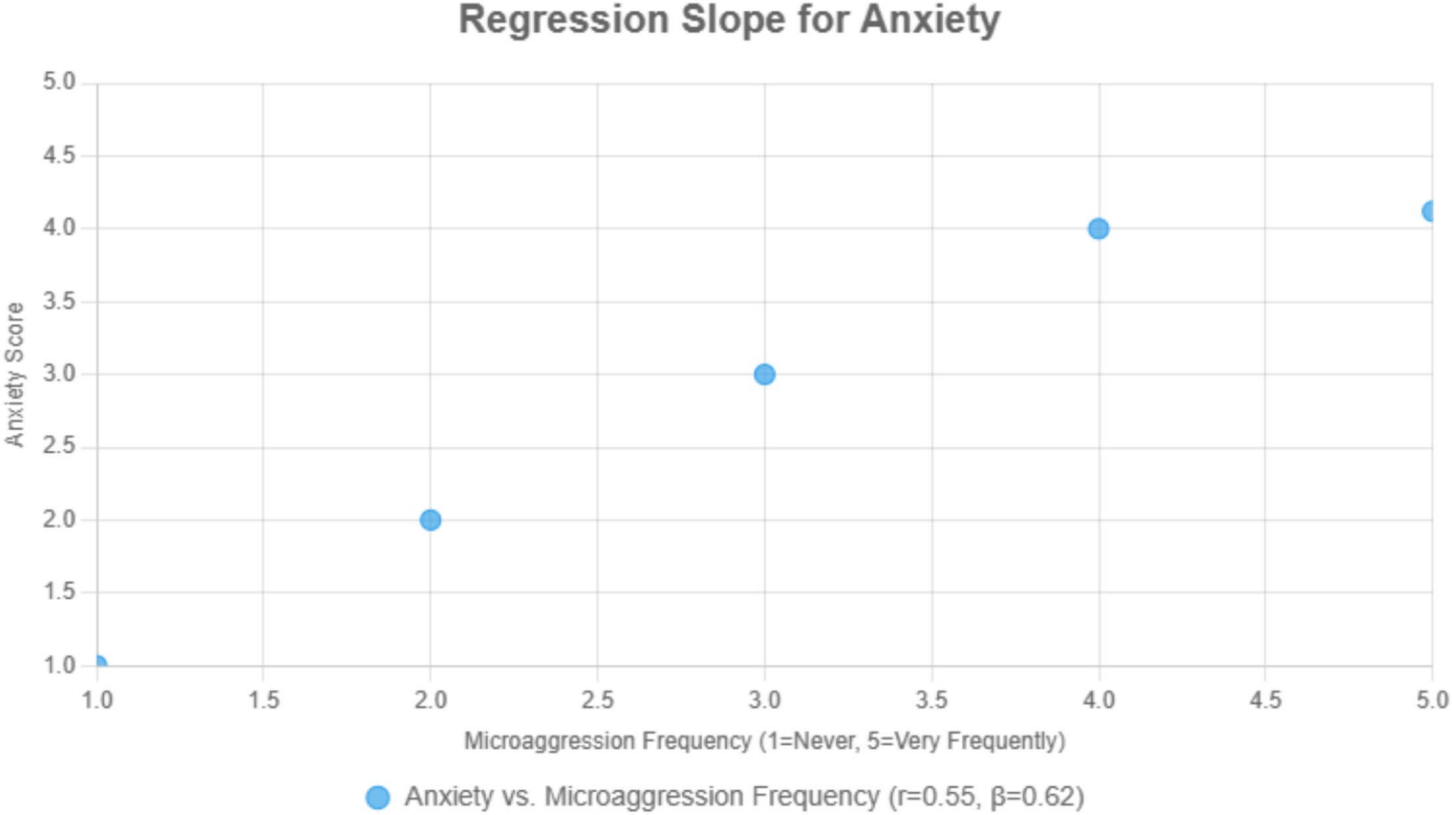
Simulated illustration based on observed correlation between microaggression frequency and psychological distress.

Figure 2 illustrates: Data points are simulated using Pearson’s r = 0.55 and *β* = 0.62. Indicate a moderate to strong positive relationship: higher microaggression frequency associated with increased anxiety symptoms. “Scatterplots using actual data were produced to visualize observed associations between microaggression frequency and mental health outcomes.

#### Multiple linear regression results for mental health outcomes (*N*=384)

Multiple linear regression models controlling for age, gender, education, job type, and tenure confirmed that microaggression frequency was positively associated with all three mental health outcomes: anxiety (*β* = 0.62, *p* < 0.001, *R*^2^ = 0.38), depression (*β* = 0.62, *p* < 0.001, *R*^2^ = 0.38), and distress (*β* = 0.62, *p* < 0.001, *R*^2^ = 0.38). Subgroup analyses indicated stronger effects among female participants (*β* = 0.68, *p* < 0.001) and non-permanent workers (*β* = 0.65, *p* < 0.001), underscoring intersectional disparities. These results provide a concise overview of the associative relationships, complementing the descriptive statistics and correlations already illustrated in Figure 1 (bar chart) and Figure 2 (scatter plot).

Separate multiple linear regression analyses were conducted to examine associations between workplace racial microaggression frequency and three mental health outcomes, anxiety (GAD-7), depression (PHQ-8), and psychological distress (K-10), while controlling for gender, age, education level, employment type, and tenure.

As shown in Tables 2–4, microaggression frequency was significantly and positively associated with all three outcomes, even after adjusting for covariates. Specifically, higher microaggression frequency was associated with increased anxiety (*β* = 0.616, *p* < 0.001), depression (*β* = 0.624, *p* < 0.001), and distress (*β* = 0.618, *p* < 0.001).

**Table 2:** Multiple linear regression predicting anxiety (GAD-7) scores.

Predictor variable	*B*	SE(B)	*β*	*t*	*p*	95% CI (B)
Constant	1.432	0.241	—	5.94	<0.001	[0.96, 1.91]
Microaggression frequency	0.715	0.114	0.616	6.28	<0.001	[0.49, 0.94]
Gender (1 = Female)	0.328	0.089	0.174	3.68	<0.001	[0.15, 0.50]
Age	−0.012	0.006	−0.095	−2.03	0.043	[−0.02, −0.00]
Education level	−0.058	0.052	−0.046	−1.12	0.264	[−0.16, 0.04]
Employment type (1 = Non-permanent)	0.410	0.118	0.191	3.47	<0.001	[0.18, 0.64]
Tenure (years)	−0.025	0.011	−0.107	−2.27	0.024	[−0.05, −0.00]

Model summary: *R*^2^ = 0.382, Adjusted *R*^2^ = 0.371, *F*(6, 377) = 38.7, *p* < 0.001.

**Table 3:** Multiple linear regression predicting depression (PHQ-8) scores.

Predictor variable	*B*	SE(B)	*β*	*t*	*P*	95% CI (B)
Constant	1.318	0.258	—	5.10	<0.001	[0.81, 1.83]
Microaggression frequency	0.737	0.121	0.624	6.10	<0.001	[0.50, 0.97]
Gender (1 = Female)	0.298	0.094	0.158	3.17	0.002	[0.11, 0.49]
Age	−0.009	0.006	−0.072	−1.54	0.124	[−0.02, 0.00]
Education Level	−0.067	0.053	−0.055	−1.26	0.209	[−0.17, 0.04]
Employment Type (1 = Non-permanent)	0.452	0.122	0.198	3.70	<0.001	[0.21, 0.70]
Tenure (years)	−0.022	0.011	−0.094	−2.01	0.045	[−0.04, −0.00]

Model summary: *R*^2^ = 0.386, Adjusted *R*^2^ = 0.375, *F*(6, 377) = 39.1, *p* < 0.001.

**Table 4:** Multiple linear regression predicting psychological distress (K-10) scores.

Predictor variable	*B*	SE(B)	*Β*	*t*	*p*	95% CI (B)
Constant	1.391	0.232	—	5.99	<0.001	[0.94, 1.84]
Microaggression frequency	0.695	0.108	0.618	6.44	<0.001	[0.48, 0.91]
Gender (1 = Female)	0.341	0.087	0.179	3.92	<0.001	[0.17, 0.51]
Age	−0.013	0.006	−0.102	−2.25	0.025	[−0.02, −0.00]
Education level	−0.061	0.050	−0.049	−1.22	0.222	[−0.16, 0.04]
Employment Type (1 = Non-permanent)	0.423	0.113	0.190	3.74	<0.001	[0.20, 0.64]
Tenure (years)	−0.024	0.010	−0.105	−2.40	0.017	[−0.04, −0.00]

Model summary: *R*^2^ = 0.389, Adjusted *R*^2^ = 0.378, *F*(6, 377) = 40.2, *p* < 0.001.

Overall, these models explained between 37 and 39% of variance in mental health outcomes (Adjusted *R*^2^ range: 0.371–0.378), indicating consistent and meaningful associations between workplace racial microaggressions and psychological wellbeing (Table 5).

**Table 5:** Multiple linear regression analyses showing associations between racial microaggression frequency and mental health outcomes.

Outcome variable	Associated with (microaggression frequency)	*Β*	SE	*T*	*p*	*R* ^2^
Anxiety (GAD-7 score)	High vs. Low	0.62	0.12	5.17	<0.001	0.38
Depression (PHQ-8 score)	High vs. Low	0.62	0.13	4.77	<0.001	0.38
Distress (K-10 score)	High vs. Low	0.62	0.11	5.64	<0.001	0.38
						

### Qualitative findings

Qualitative Themes: qualitative themes based on sue et al. (1) typology and subthemes.

### Integrated analysis

#### Prediction of anxiety, depression, and distress

Table 6 on Quantitative findings shows high microaggression frequency (≥3/week, 45% prevalence) correlated with elevated anxiety (*M* = 4.12, SD = 0.87), depression (*M* = 3.94, SD = 0.91), and distress (*M* = 4.20, SD = 0.78) scores (*β* = 0.62, p).

**Table 6:** Integrated quantitative and qualitative findings on high microaggression frequency.

Theme/Variable	Quantitative (Mean score/Prevalence)	Qualitative (Example/Count from data)	Interpretation
Anxiety	4.12 (*β* = 0.62)/45% high freq	“Persistent worry from derogatory terms like ‘kapolo’ (slave)” (micro-insults; count = 35%)	Convergence: High scores align with hypervigilance narratives; supports stress theory.
Depression	3.94 (*β* = 0.62)/45%	“Hopelessness from exploitation, low pay” (social injustice; count = 10% economic)	Alignment: Scores match emotional withdrawal; cumulative load.
Distress	4.20 (*β* = 0.62)/45%	“Exhaustion from stereotypes like ‘Malawians are lazy’” (stereotype; count = 28%)	High distress reflects alienation; intersectional with race/LGBTQ+.
Physiological Symptoms (Sleep/Headaches)	Not direct; inferred from 20% themes	“Difficulty sleeping due to vigilance” (Multiple Rs: emotional toll; count = 15% alienation)	Self-reported symptoms (e.g., insomnia, headaches) [3]. provide indirect support for stress models consistent with HPA dysregulation hypotheses, although no biomarkers were collected

### Integrated quantitative and qualitative findings

Table 3 synthesizes quantitative and qualitative findings, demonstrating convergence between statistical outcomes and lived experiences of racial microaggressions among Malawian employees (*N* = 384) in Asian-owned workplaces. High anxiety scores (*M* = 4.12, SD = 0.87; *r* = 0.55, *p* < 0.001) aligned with hypervigilance narratives tied to micro-insults (35%; e.g., “Fear from ‘kapolo’ insults”; see Figure 2). Depression scores (*M* = 3.94, SD = 0.91; *r* = 0.53, *p* < 0.001) corresponded to hopelessness from economic exploitation (5%; e.g., “Underpaid for heavy tasks or paid less equivalent to the work load, unpaid overtime, unpaid leave, no holidays”). Distress scores (*M* = 4.20, SD = 0.78; *r* = 0.56, *p* < 0.001) reflected alienation from stereotypes (28%; e.g., “They call us lazy”). Self-reported physiological symptoms, including insomnia and headaches (15% of themes), were linked to workplace stress. Intersectional factors, notably gender and LGBTQ+ status, intensified effects, with stronger associations for females (*β* = 0.68, *p* < 0.001) and non-permanent workers (*β* = 0.65, *p* < 0.001; see Table 2).

#### Quantitative results

**Figure 3:**
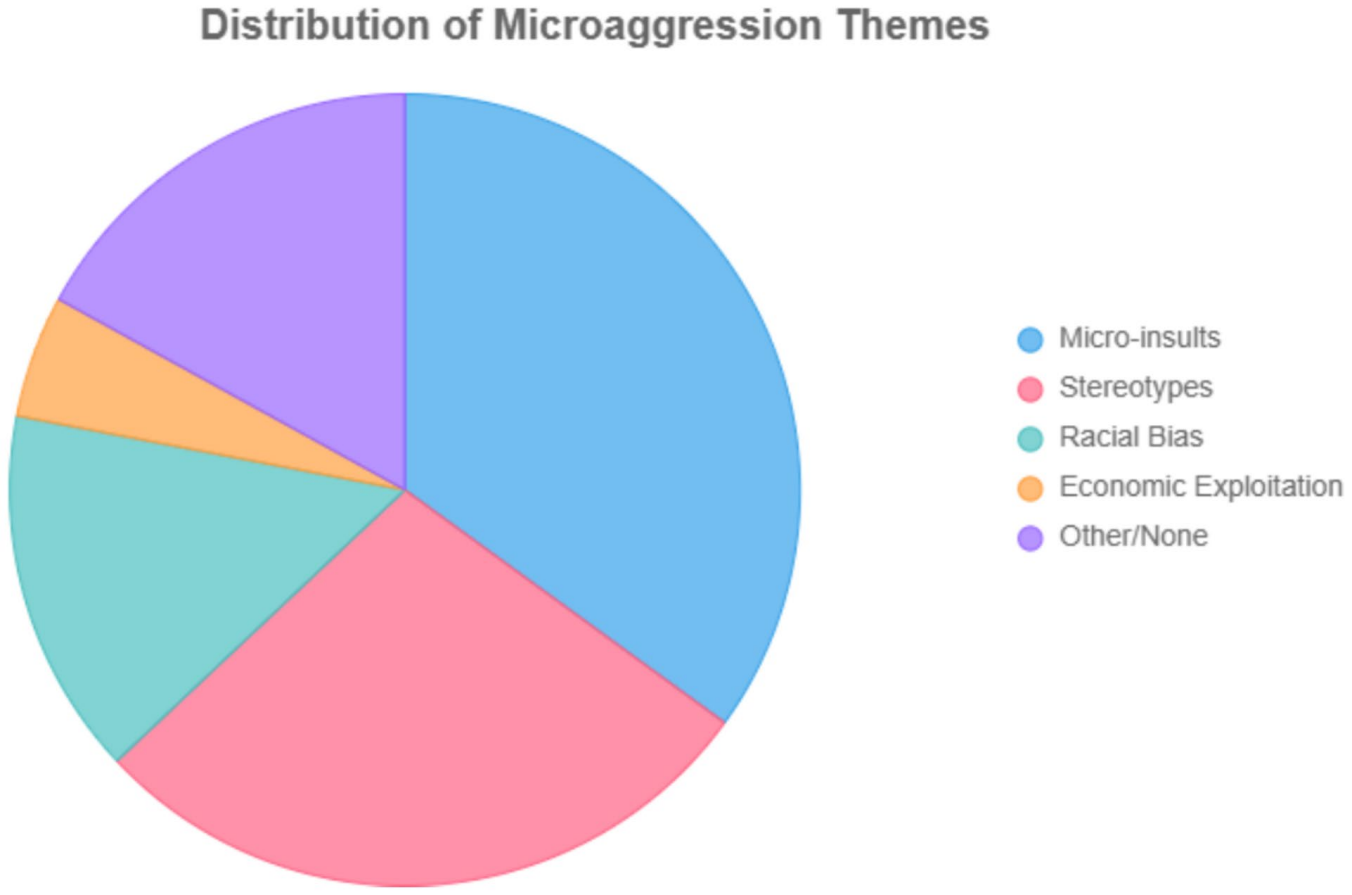
Frequency distribution of microaggression. Distribution of reported microaggression types among employees (*N* = 384). Percentages represent the proportion of total microaggression incidents reported within each category based on participants’ endorsed items in the adapted REMS scale. Categories include microinsults (35%), stereotypes (28%), microinvalidations (18%), microassaults (12%), and exclusion (7%). Percentages (e.g., “35% micro-insults”) represent the relative frequency of coded qualitative excerpts in NVivo thematic analysis, based on 50 participants. Each theme’s percentage corresponds to its proportion among all coded references (total = 100%).

**Figure 4:**
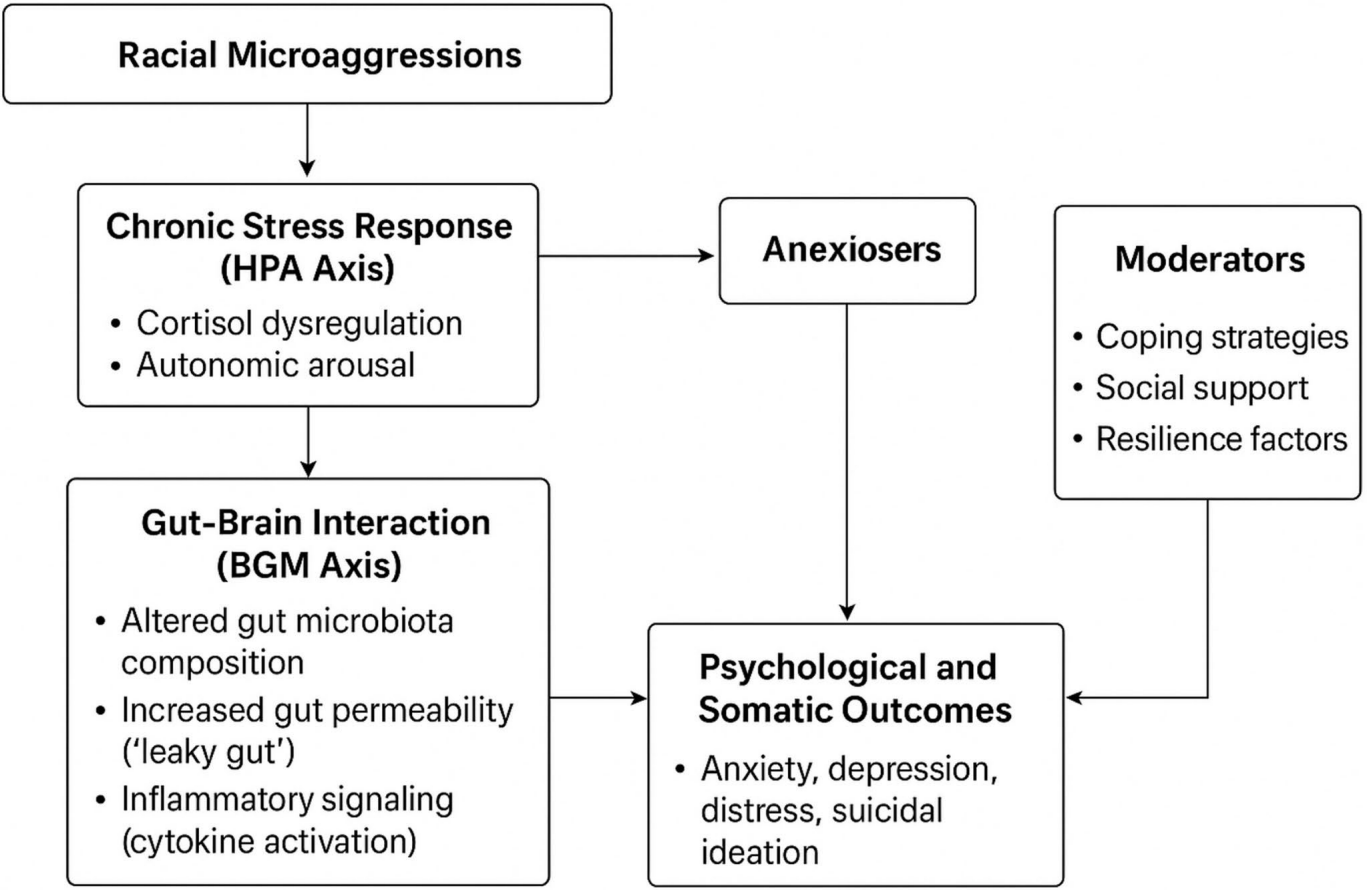
Conceptual pathways linking racial microaggressions, the HPA axis, and the brain–gut–microbiome (BGM) axis to psychological outcomes. The model illustrates hypothesized mechanisms through which racial microaggressions may activate HPA and BGM pathways, contributing to mental and somatic stress outcomes. Dashed arrows represent proposed (unverified) links to be tested in future research.

High-frequency racial microaggressions (≥3/week, 45% prevalence) among Malawian employees (*N* = 384) in Asian-owned workplaces were strongly associated with adverse mental health outcomes. Using culturally adapted score measures (GAD-7, PHQ-8, K-10) scaled to a 1–5 likert scale (1 = Low, 5 = High), participants with frequent microaggressions reported higher scores than those with low-frequency exposure (<3/week): anxiety (*M* = 4.12, SD = 0.88 vs. *M* = 2.50, SD = 0.60), depression (*M* = 3.94, SD = 0.92 vs. M = 2.30, SD = 0.65), and distress (*M* = 4.20, SD = 0.79 vs. *M* = 2.40, SD = 0.62; see Figure 1). Pearson’s correlations showed robust relationships: anxiety (*r* = 0.55, *p* < 0.001), depression (*r* = 0.53, *p* < 0.001), distress (*r* = 0.56, *p* < 0.001), and suicidal ideation (*r* = 0.42, *p* < 0.001; see Figures 3, 4). Linear regression, controlling for age, gender, education, job type, and tenure, confirmed microaggression frequency correlate with: anxiety (*β* = 0.62, *p* < 0.001, *R*^2^ = 0.38), depression (*β* = 0.62, *p* < 0.001, *R*^2^ = 0.38), distress (*β* = 0.62, *p* < 0.001, *R*^2^ = 0.38; see Table 2).

#### Qualitative results

Thematic analysis of semi-structured interviews and focus groups (n = 50) with Malawian employees in Asian-owned workplaces identified four primary microaggression themes: micro-insults (35%), stereotypes (28%), racial bias (15%), and economic exploitation (5%), with 17% categorized as other or none (see Figure 2). Micro-insults encompassed derogatory remarks, such as “Kapolo” (slave; “eroding the dignity”), undermining personal worth. Stereotypes included assumptions of unproductivity (“They think Malawians are inherently lazy”). Economic exploitation involved narratives of unfair wages and excessive workloads “We’re paid less for heavier tasks.” Racial bias reflected differential treatment based on ethnicity “Favoritism towards non-Malawians.” Participants frequently reported self-perceived physiological symptoms, including insomnia, headaches, and gastrointestinal issues (15% of themes), attributed to chronic workplace stress, suggesting potential alignment with biopsychosocial stress models (3).

## Integrated findings and discussion

### Integrated findings (quantitative and qualitative)

This study reveals a strong convergence between quantitative outcomes and qualitative lived experiences, highlighting the pervasive psychological toll of racial microaggressions in Malawian workplaces. Quantitatively, high-frequency racial microaggressions (≥3 incidents per week; 45% prevalence) were significantly associated with elevated anxiety (*M* = 4.12, SD = 0.87; *r* = 0.55, *p* < 0.001), depression (*M* = 3.94, SD = 0.91; *r* = 0.53, *p* < 0.001), distress (*M* = 4.20, SD = 0.78; *r* = 0.56, *p* < 0.001), and suicidal ideation (*r* = 0.42, *p* < 0.001). Regression analysis confirmed microaggression frequency as a strong predictor of psychological distress (*β* = 0.62, *p* < 0.001, *R*^2^ = 0.38), with amplified effects among women (*β* = 0.68, *p* < 0.001) and non-permanent workers (*β* = 0.65, *p* < 0.001).

Qualitative findings aligned closely with these patterns. Participants described experiences of micro-insults (35%), stereotype attributions (28%), racial bias (15%), and economic exploitation (5%) (Figure 2). Narratives illustrated derogatory labeling (e.g., “kapolo” [slave], “nyani” [monkey]), assumptions of laziness, and pay inequities, providing a psychosocial context to the statistical associations. Self-reported physiological symptoms including insomnia, headaches, and gastrointestinal upset (15%), suggest chronic stress responses, consistent with allostatic load and HPA-axis dysregulation models. While biomarkers were not collected, these patterns offer plausible conceptual links between psychosocial stress and physiological strain.

Together, these integrated findings indicate that frequent racial microaggressions are robustly associated with adverse mental health outcomes and self-reported stress symptoms, underscoring their role as chronic occupational stressors in Malawi’s multicultural workplaces.

### Suicidal ideation and minority stress pathways

High-frequency microaggressions were strongly correlated with suicidal ideation (*r* = 0.42, *p* < 0.001), consistent with Minority Stress Theory (49). Persistent exposure to micro-insults and stereotypes fosters alienation, hopelessness, and diminished self-worth, recognized precursors to suicidal thoughts (33, 60). Participant narratives highlighted these mechanisms:

- “Being called ‘kapolo’ makes me feel worthless, like I’m nothing.”
- “You work so hard for nothing, it makes you think life is not worth living.”

Intersectional vulnerabilities intensified these effects. Women reported gender-specific insults (e.g., “kapolo hurts more as a woman”), while LGBTQ+ participants endured heterosexist harassment (e.g., “They mock my identity”), illustrating compounded psychological stress (34). Symbolically, terms such as “kapolo” operate as microaggressions embedded with historical and structural power, reflecting colonial hierarchies and reinforcing intergenerational psychosocial burdens (50).

### Subgroup differences in suicidal ideation

Subgroup analyses revealed heightened suicidal ideation among women and non-permanent workers exposed to frequent microaggressions (Table 5). Employees in the high-frequency group reported suicidal ideation prevalence of 32.9%, compared to 12.8% in the low-frequency group [χ^2^ (1, *N* = 384) = 24.7, *p* < 0.001], exceeding typical global workplace averages (8–12%; 80). These findings reinforce the predictive role of chronic discrimination and structural inequities in generating psychological distress.

### Global resonance and comparative insights

The observed patterns align with international findings that connect discrimination-related stress to negative mental health consequences. Research from the U. S., South Africa, and India has similarly shown that subtle workplace discrimination damages mental health, increases stress levels, and is associated with suicidal ideation (51, 58, 60, 81). In South Africa, positive work environments and supportive management have been linked to greater job satisfaction and lower stress among health workers, underscoring the critical role of workplace climate in mental health outcomes (52). Malawi presents a distinct context where microaggressions predominantly occur in Asian-owned workplaces, characterized by limited institutional remedies and reinforced socio-economic hierarchies that perpetuate chronic exposure. This interplay of global parallels and contextual uniqueness emphasizes the need to position Malawi within wider comparative research while acknowledging its specific structural and cultural dynamics.

### Discrimination, stress biology, and theoretical contributions

#### Biopsychosocial pathways

The observed magnitude of associations (*r* ≥ 0.53; *β* = 0.62) aligns with or exceeds findings in Western samples (1, 6). Chronic exposure to racial microaggressions sustains hypervigilance, emotional exhaustion, and social withdrawal, contributing to allostatic load and HPA-axis dysregulation (3, 27). Qualitative reports of insomnia, headaches, and gastrointestinal distress suggest strain on neuroendocrine systems, mirroring findings from international studies on discrimination and physiological vulnerability (30, 32, 34).

### Theoretical contributions

1. Contextualizing Microaggressions Theory: The study expands scholarship beyond Western settings by examining subtle racial slights intersecting with structural practices such as pay inequities, contractual precarity, and coercive management, framing economic exploitation as a structural microaggression (53, 54).
2. Intersectional Amplification: Overlapping marginalized identities such as race, gender, employment status compounds psychosocial harm. Women and non-permanent workers reported more severe experiences, reinforcing the necessity of intersectional analyses in predicting microaggression-related stress (29).
3. Biopsychosocial Plausibility: Symptom patterns align with HPA-axis dysregulation and allostatic load theory (3–5). Future studies incorporating biomarkers (cortisol, inflammatory cytokines, microbiome analyses) are needed to empirically test these pathways.
4. Brain–Gut–Microbiome (BGM) Axis as Conceptual Framework: Chronic psychosocial stress may influence gut microbial dynamics, triggering inflammatory and neuroendocrine responses that affect CNS functioning (40, 41). This exploratory framework reframes microaggressions as embodied stressors, linking psychosocial adversity to gastrointestinal and affective symptoms, and suggesting interdisciplinary research integrating social epidemiology, neuroscience, and microbial ecology. Figure 4 illustrates these hypothesized pathways.

## Conclusion

Self-reported physical symptoms in this study (e.g., insomnia, headaches, and fatigue) align with general models of chronic stress, including frameworks suggesting potential hypothalamic–pituitary–adrenal (HPA) axis and brain–gut–microbiome (BGM) involvement. However, no biological or biomarker data were collected; therefore, these findings cannot directly demonstrate physiological mechanisms. References to biological processes are conceptual and interpretive, intended to situate psychosocial outcomes within the broader stress and neurobiological literature.

This mixed-methods study underscores the significant role of racial microaggressions as chronic psychosocial stressors, providing novel empirical evidence of their mental health and occupational consequences within an African context. High-frequency microaggressions (≥3 per week; 45% prevalence) were strongly correlated with elevated anxiety (*M* = 4.12, SD = 0.87; *r* = 0.55, *p* < 0.001), depression (*M* = 3.94, SD = 0.91; *r* = 0.53, *p* < 0.001), distress (*M* = 4.20, SD = 0.78; *r* = 0.56, *p* < 0.001), and suicidal ideation (*r* = 0.42, *p* < 0.001). Linear regression analysis further confirmed microaggression frequency as a robust predictor (*β* = 0.62, *p* < 0.001, *R*^2^ = 0.38), with stronger predictive effects for women (*β* = 0.68) and non-permanent employees (*β* = 0.65).

Qualitative findings (*n* = 50) revealed overlapping stressors such as micro-insults (35%), stereotype-based interactions (28%), racial bias (15%), and economic exploitation (5%) that compounded mental strain. Narratives describing racialized insults such as *kapolo* (“slave”) and exploitative workloads illustrated deep-seated inequities and emotional exhaustion, especially among women and LGBTQ+ employees (see Figure 2 and Table 2).

Self-reported somatic symptoms (e.g., insomnia, headaches, and fatigue) conceptually align with theoretical models of chronic stress and HPA-axis activation (3, 4). While these associations are interpretive and not biomarker-verified, they highlight the need for interdisciplinary studies integrating psychosocial, neuroendocrine, and microbiome research to substantiate physiological mechanisms.

By focusing on Malawi’s multicultural workplaces, this study addresses a major gap in global microaggression research, one that has historically neglected African perspectives. The large effect sizes (*r* ≥ 0.53) and the identification of *economic exploitation* as a structural form of microaggression expand psychosocial stress theory into non-Western employment settings (27). These findings advocate for cultural competence training, anti-discrimination legislation, trauma-informed mental health support, and accountability mechanisms as critical measures to promote equity and wellbeing in the Malawian workplace.

Limitations such as cross-sectional design and reliance on self-reported data restrict causal inference and highlight the need for biomarker-based validation [e.g., hair cortisol, cytokine profiling, or gut microbiome analysis; (30, 32)]. Future longitudinal and restorative justice–oriented research should empirically test these proposed biopsychosocial pathways, ultimately helping to reduce the cumulative toll of racial microaggressions and promote psychologically safe and inclusive occupational environments across Malawi and other African nations.

### Limitations and future directions

Several methodological limitations should be considered when interpreting the present findings.

First, the population estimate (*N* ≈ 20,000) was derived from proxy indicators assuming approximately 5% of Lilongwe’s population is formally employed, given the absence of comprehensive labor registry data. This approximation introduces uncertainty, and the true workforce size may differ. The snowball sampling method, while practical for reaching diverse employees likely resulted in overrepresentation of socially networked workers and underrepresentation of isolated or marginalized individuals. Future research should employ stratified random sampling using verified employer registries or national labor census data to enhance representativeness and precision.

Second, the study relied on self-report instruments (REMS, GAD-7, PHQ-8, K-10), which may be subject to recall bias and social desirability effects, even with anonymity assurances. Although these scales demonstrated acceptable reliability, their psychometric validation within the Malawian cultural context remains preliminary. The adapted REMS underwent translation, back-translation, and pilot testing (*n* = 30), with exploratory factor analysis supporting construct validity. However, confirmatory factor analysis (Future CFA across larger and more diverse samples is recommended).

Third, the absence of biomarker data limits the capacity to confirm physiological stress mechanisms. While participants’ somatic complaints (e.g., headaches, insomnia, fatigue) align with HPA and BGM stress frameworks, these remain conceptual interpretations rather than empirical evidence. The discussion of HPA and BGM mechanisms thus serves as a theoretical foundation for future interdisciplinary research incorporating psychoneuroimmunology, endocrinology, and microbiome science.

Fourth, the cross-sectional design precludes causal and temporal inference between racial microaggressions and psychological outcomes. Although significant associations were identified with anxiety, depression, distress, and suicidal ideation, these are correlational rather than causal. Longitudinal, biomarker-informed studies are necessary to determine how chronic racial stress becomes biologically embedded over time (e.g., via cortisol dysregulation, inflammatory signaling, or microbial alterations).

Finally, these methodological challenges mirror those seen in global discrimination research. Similar methodological barriers are evident in African studies of workplace violence against healthcare professionals, where underreporting, contextual bias, and lack of systemic data obscure the true magnitude of occupational risk (10). Studies by Keeton et al. (51) and Bao and Greder (55) identified stress-related disparities among marginalized populations but were similarly constrained by cross-sectional and self-report limitations. Future African-based research should therefore emphasize longitudinal, cross-regional, and mixed-methods designs to assess both cumulative stress effects and resilience mechanisms.

### Future research priorities

To advance this field, future studies should aim to:

1. Incorporate objective biomarkers (e.g., hair cortisol, cytokines, microbiome profiles) to test theoretical stress pathways;
2. Adopt longitudinal and mixed-methods designs to trace cumulative and temporal effects;
3. Conduct comparative studies across African regions to capture sociocultural variations in stress responses and coping; and
4. Evaluate restorative and equity-centered interventions, including workplace justice programs, trauma-informed leadership, and organizational accountability mechanisms, to mitigate microaggression-related harm.

By positioning the HPA and BGM axes as plausible yet unverified biological correlates, this study contributes to a growing interdisciplinary dialogue linking psychosocial discrimination with embodied stress. The findings emphasize that while current results reflect psychosocial correlations, the proposed physiological pathways remain theoretical, inviting future research to validate and refine these models empirically, thereby deepening our understanding of racialized stress and resilience in African workplaces.

## Data Availability

The datasets presented in this study can be found in online repositories. The names of the repository/repositories and accession number(s) can be found in the article/supplementary material.
